# Supplementary material for: Brainstem network dynamics underlying the encoding of bladder information
Source: eLife. 2017 Dec 4;6:e29917. doi: 10.7554/eLife.29917 (PMC5714501; doi:10.7554/eLife.29917)
Supplement: Transparent reporting form [file elife-29917-transrepform.docx]

***eLife’s* transparent reporting form**

We encourage authors to provide detailed information *within their submission* to facilitate the interpretation and replication of experiments. Authors can upload supporting documentation to indicate the use of appropriate reporting guidelines for health-related research (see [EQUATOR Network](http://www.equator-network.org/%20)), life science research (see the [BioSharing Information Resource](https://biosharing.org/)), or the [ARRIVE guidelines](http://www.plosbiology.org/article/info:doi/10.1371/journal.pbio.1000412) for reporting work involving animal research. Where applicable, authors should refer to any relevant reporting standards documents in this form.

If you have any questions, please consult our Journal Policies and/or contact us: [editorial@elifesciences.org](mailto:editorial@elifesciences.org).

**Sample-size estimation**

- You should state whether an appropriate sample size was computed when the study was being designed
- You should state the statistical method of sample size computation and any required assumptions
- If no explicit power analysis was used, you should describe how you decided what sample (replicate) size (number) to use

Please outline where this information can be found within the submission (e.g., sections or figure legends), or explain why this information doesn’t apply to your submission:

All the sample sizes (#animals, #neurons) were mentioned at the beginning of each Result subsection. For Barrington’s nucleus data (page 5, line 88), for LC data (page 7, line 157; page 8, lines 171-172), for LC-PFC data, (Page 10, lines 206-207). Estimates of sample size were not computed when the study was designed because there was no basis to estimate effect size (d) apriori. Additionally, it could not be known how many cells would be obtained in each animals. For Barrington’s nucleus raw neuronal data are presented for each individual cell in each of three rats (Figure 1D2). Power calculations determined retrospectively using the power table in Howell Statistical Methods for Psychology (8^th^ Edition) and the equation δ=d($\sqrt{n}$) indicated sufficient power with the n used for each rat. For the top panel power =0.95, for the middle panel power =0.87 and for the bottom panel power=0.99. LC data obtained from 51 cells from 4 rats was converted to z score where each value is subtracted from the mean and divided by the standard deviation. This is described in the Methods (Page 19, line 409-411).

**Replicates**

- You should report how often each experiment was performed
- You should include a definition of biological versus technical replication
- The data obtained should be provided and sufficient information should be provided to indicate the number of independent biological and/or technical replicates
- If you encountered any outliers, you should describe how these were handled
- Criteria for exclusion/inclusion of data should be clearly stated
- High-throughput sequence data should be uploaded before submission, with a private link for reviewers provided (these are available from both GEO and ArrayExpress)

Please outline where this information can be found within the submission (e.g., sections or figure legends), or explain why this information doesn’t apply to your submission:

**There were three types of experiments described in this paper.**

1) **Activity of neurons in the LC**
2) **Activity of neurons in the BN**
3) **Spectral measures from simultaneous LC and mPFC field potential recordings.**

- **The first biological replicate is the number of animals**
  - **Exp1-LC n=4;**
  - **Exp2- BN n=3;**
  - **Exp3- mPFC-LC measures n=3**
- **The second level of biological replicates is number of neurons**
  - **Exp1 –LC n=51 neurons**
  - **Exp2-BN = =36 neurons**
  - **Exp3 – no replication here because only field potentials were recorded**
- **The technical replication in our case was the number of micturition cycles from which data were obtained for each animal (page 7, line 138, page 8, line 172 and page 10, line 206-207). Additionally, activity from different cells recorded on multiple channels in the same subject provided technical replication.**
  - **1-4 micturition cycles were analyzed per animal. This counts as a technical replication of the same biological sample.**

**All data from discriminated units recorded within the histologically identified appropriate region (LC or Barrington’s nucleus ) were used. This is now stated in the Methods (page 19, line 424).**

**Statistical reporting**

- Statistical analysis methods should be described and justified
- Raw data should be presented in figures whenever informative to do so (typically when N per group is less than 10)
- For each experiment, you should identify the statistical tests used, exact values of N, definitions of center, methods of multiple test correction, and dispersion and precision measures (e.g., mean, median, SD, SEM, confidence intervals; and, for the major substantive results, a measure of effect size (e.g., Pearson's r, Cohen's d)
- Report exact p-values wherever possible alongside the summary statistics and 95% confidence intervals. These should be reported for all key questions and not only when the p-value is less than 0.05.

Please outline where this information can be found within the submission (e.g., sections or figure legends), or explain why this information doesn’t apply to your submission:

For Barrington’s nucleus data the raw data are provided for individual neurons in three different rats in Figure 1D2. Paired samples t-tests were used to evaluate differences between the firing rate in the BN pre- and post- micturition (p-values reported in the Fig1D2).

Z-scores were obtained for all LC firing rate values in Fig 2. This method is described in Methods on page 19, lines 409-411.

For Power spectra or coherence comparisons shown in Figure 4, two way repeated measures ANOVA were used and the values are provided in the figure legend, page 11, lines 230-231.

Raw data examples were provided in every figure (Fig 1C1,C2, D1 ; Fig 2A ; Fig3C ; Fig4A,B)

Mean +/- SEM were displayed

(For large datasets, or papers with a very large number of statistical tests, you may upload a single table file with tests, Ns, etc., with reference to sections in the manuscript.)

**Group allocation**

- Indicate how samples were allocated into experimental groups (in the case of clinical studies, please specify allocation to treatment method); if randomization was used, please also state if restricted randomization was applied
- Indicate if masking was used during group allocation, data collection and/or data analysis

Please outline where this information can be found within the submission (e.g., sections or figure legends), or explain why this information doesn’t apply to your submission:

The experimental design was a within subject design so that there was no group allocation Whether rats were used for LC or Barrington’s nucleus recordings was dependent on post-hoc determination of the electrode site, i.e., whether the recording was histologically identified in the LC or Barrington’s nucleus. There was no masking during data collection or analysis given the experimental design.

**Additional data files (“source data”)**

- We encourage you to upload relevant additional data files, such as numerical data that are represented as a graph in a figure, or as a summary table
- Where provided, these should be in the most useful format, and they can be uploaded as “Source data” files linked to a main figure or table
- Include model definition files including the full list of parameters used
- Include code used for data analysis (e.g., R, MatLab)
- Avoid stating that data files are “available upon request”

Please indicate the figures or tables for which source data files have been provided:
